# Acute and Past Common Lacrimal Canalicular Lacerations: A Report of Two Cases

**DOI:** 10.7759/cureus.53420

**Published:** 2024-02-01

**Authors:** Shinjiro Kono, Motohiro Kamei

**Affiliations:** 1 Department of Ophthalmology, Aichi Medical University Hospital, Nagakute, JPN

**Keywords:** dacryocystorhinostomy, canalicular stent insertion, laceration, trauma, common canaliculus

## Abstract

Lacrimal canalicular lacerations can be caused by trauma to the ocular adnexa, such as a penetrating or blunt injury. Only a few cases involving the common canaliculus or lacrimal sac have been reported, and only a few reports have described the detailed course of treatment. Here, we report an acute case of common lacrimal canalicular laceration and a case of a previous common canalicular laceration. The acute case was treated with a bicanalicular stent directly inserted into the nasolacrimal duct. The case with a previous common canalicular laceration was treated with external dacryocystorhinostomy combined with monocanalicular stent insertion. These treatments may be appropriate for the initial surgery and can be selected before performing conjunctivodacryocystorhinostomy.

## Introduction

Lacrimal canalicular lacerations can be caused by trauma to the ocular adnexa, such as a penetrating or blunt injury. Canalicular disruption complicates 16-37% of routine eyelid lacerations [1-3]. Canalicular anastomosis combined with stent intubation has been used to repair canalicular lacerations. Bicanalicular lacerations are conventionally treated with bicanalicular stents [4-6], and recently, the successful reconstruction of a bicanalicular laceration to the lacrimal sac with two monostents has been reported [7,8]. Old laceration cases or more complicated cases are sometimes treated with dacryocystorhinostomy in combination [9-13].

Cases of monocanalicular and bicanalicular lacerations are frequently reported. However, only a few cases involving the common canaliculus or lacrimal sac have been reported, with little information on the detailed course of treatment [6,9,10]. In this study, we present two cases: an acute case of common lacrimal canalicular laceration and a case of a past common canalicular laceration; furthermore, we indicate available treatment options.

## Case presentation

Case 1

**Figure 1:**
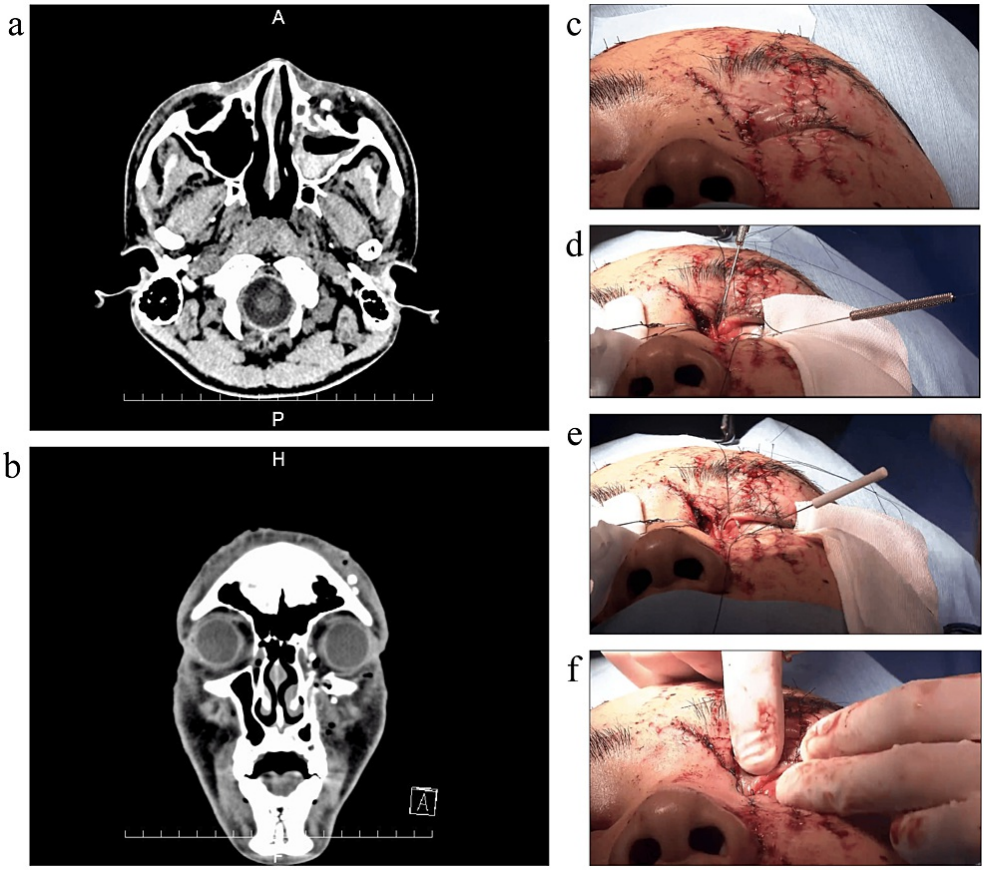
Case 1 (a, b) Preoperative CT images show depression of the anterior aspect of both maxillary sinuses and bilateral zygomatic fractures as well as high intensity areas that appear to be multiple glass fragments; (c–f) Photo of the face taken during the surgery; (c) Before the incision. The wound was sutured in the emergency room the previous day; (d) The sutured wound of the medial canthus is opened. A bougie is inserted through the upper and lower lacrimal canaliculi into the proximal cut surface to confirm the presence of a hard stop; (e, f) A bicanalicular stent is inserted from the upper and lower lacrimal puncta toward the nasolacrimal duct, followed by wound closure Written informed consent for the publication of facial photographs was obtained from the patient.

A 19-year-old male was riding straight ahead on a motorcycle when he collided with a car and became trapped in its rear window. The patient was transported to the emergency department of our hospital with multiple facial lacerations. Computed tomography (CT) imaging revealed depression of the anterior aspect of both maxillary sinuses and bilateral zygomatic fractures (Figure 1a, 1b). Contusion wounds were found on the right forehead, right upper and lower eyelids, left upper and lower eyelids, and medial canthus.

Emergency and plastic surgeons collaborated to suture the wounds in the emergency room (Figure 1c). At that time, left lacrimal canalicular laceration was suspected based on the location of the wound and lacrimal syringing examination. The day after suturing, the surgeons consulted the surgical team regarding the canalicular laceration. Lacrimal syringing examination revealed ipsilateral retrograde flow in the upper and lower lacrimal ducts, with leakage from the wound. Based on the location of the wound and CT findings, a common lacrimal canalicular laceration was suspected. Reconstruction of the lacrimal canaliculus was scheduled under local anesthesia.

The sutured wound on the medial canthus was opened. A bougie was placed through the upper and lower lacrimal canals to confirm the distal cutoff surface. The proximal cut surface of the canaliculus was examined deep on the nasal side of the incisional wound. During the search, glass fragments were identified and removed. We then found the proximal cut surface and inserted a bougie through the upper and lower lacrimal canaliculi (Figure 1d). We confirmed a hard stop on probing and inserted the bougie into the nasolacrimal duct from the upper and lower puncta without resistance. After sutures were placed on Horner's muscle, a bicanalicular stent was inserted from the upper and lower lacrimal puncta toward the nasolacrimal duct (Figure 1e). Horner's muscle around the canaliculus was sutured to the medial canthal tissue as a canalicular anastomosis to reconstruct the canaliculi. Finally, the orbicularis oculi muscle and subcutaneous tissue were sutured with 6-0 polydioxanone (PDS®; Ethicon, Inc., Raritan, New Jersey, United States), and the skin was closed with 6-0 polyvinylidene fluoride (ASFLEX®; Kono Seisakusho Co., Ltd, Chiba, Japan) (Figure 1f).

The inserted lacrimal stent was accidentally removed on the third postoperative day by the patient; however, the stent was reinserted four days later when the plastic surgeons performed open reduction and internal fixation for bilateral zygomatic fracture. When the stent was removed five months after surgery, lacrimal syringing was performed. The upper lacrimal canaliculus allowed the passage of fluid; however, the lower lacrimal canaliculus did not. The lower lacrimal canal was obstructed. The patient still had slight epiphora; however, the symptoms did not interfere with his life.

Case 2

**Figure 2:**
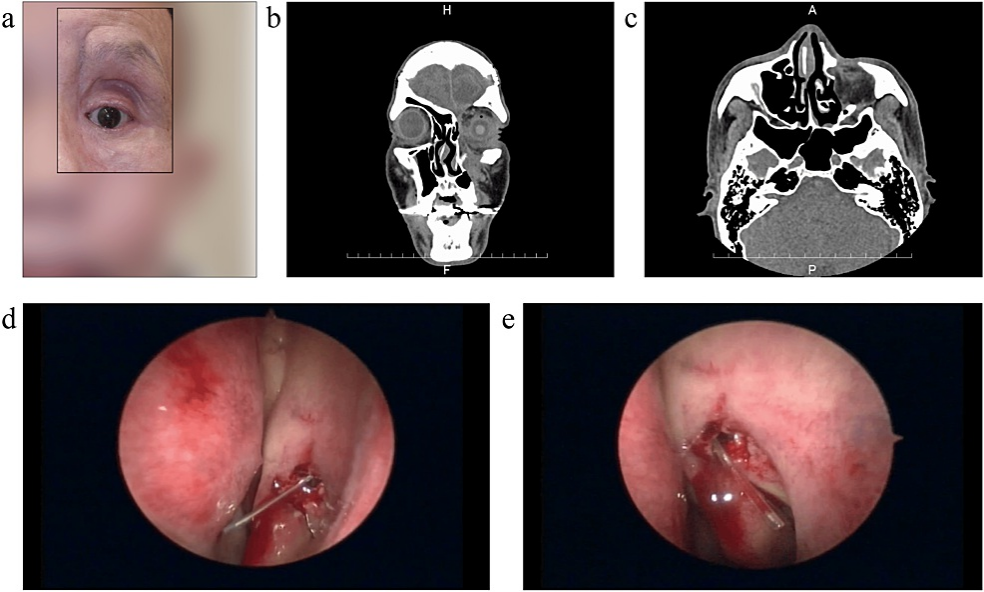
Case 2 (a) Preoperative frontal photo of the face showing the vertical scar on the medial side of the left orbit, from above the eyebrow to the cheek; (b, c) Preoperative CT images showing a bone defect in the anterior wall of the left maxillary sinus and inferior margin of the orbit, a fracture on the inferior wall of the orbit, and drooping of the intraorbital adipose tissue as well as bone defects on the lateral wall of the nasolacrimal duct; (d, e) Intraoperative image of the intranasal cavity. A monocanalicular stent is inserted into the nasal cavity through the lower lacrimal puncta and a nasal endoscope is used to confirm and adjust the stent position. Written informed consent for the publication of facial photographs was obtained from the patient.

A 59-year-old male presented to our hospital with the chief complaint of epiphora (Figure 2a). Eight months previously, he was struck on the head and face at a construction site. An open fracture of the left frontal bone and a comminuted fracture of the face, which included zygomatic, maxillary, and orbital fractures were noted. Plastic surgery and neurosurgery were performed. The plastic surgeons repaired the frontal bone fracture and removed bone fragments. The neurosurgeons removed intracerebral hematomas and treated spinal fluid leaks. Since then, he had been experiencing lacrimal symptoms but had not been informed about the lacrimal canalicular laceration. He was referred to our clinic because he was informed about the lacrimal canal injury at his doctor's visit two months earlier and desired treatment.

CT imaging revealed a bone defect in the anterior wall of the left maxillary sinus and inferior margin of the orbit, fracture of the inferior wall of the orbit, and drooping of the intraorbital adipose tissue. Bone defects were also observed in the lateral wall of the nasolacrimal duct, and soft-density areas were contiguous with the subcutaneous and maxillary sinuses, indicating inflammatory or postoperative changes (Figure 2b, 2c). Lacrimal syringing revealed ipsilateral reflux from the upper and lower canaliculi. The bougie insertion showed a soft stop upon probing.

Surgery was performed under general anesthesia, considering that the search for lacrimal canaliculi might be time-consuming given the duration since the laceration and that conjunctivo-dacryocystorhinostomy (C-DCR) would be performed if no lacrimal canaliculus was found. We opened the previous surgical scar on the medial lower eyelid, dissected the subcutaneous tissue and orbicularis oculi muscle, and reached the lacrimal fossa. We searched for the cut surface of the canaliculus toward the lateral part of the lacrimal sac but did not immediately find it. A bougie was inserted through the upper and lower lacrimal puncta. Following the lower canaliculus, while puncturing the scar tissue with a bougie, the bougie was observed to reach the lacrimal sac. A bougie was also inserted through the upper lacrimal canaliculus; however, the upper lacrimal canaliculus was not directed toward the lacrimal sac and could not reach it due to the scar.

The nasolacrimal duct was suspected to have significant traumatic changes on CT images and the bougie was confirmed in the lacrimal sac; therefore, we converted the procedure to external DCR (Ex-DCR). We detached the lacrimal sac from the orbital bone, created a bony window with an ultrasonic bone aspirator (Sonopet UST-2000™; Stryker Corporation, Kalamazoo, Michigan, United States), and performed rhinostomy. We created a nasal mucosal flap and lachrymal sac mucosal flap, sutured them, completed a bony window, and inserted a monocanalicular tube (Masterka®; FCI S.A.S. - France Chirurgie Instrumentation, Paris, France) from the lower lacrimal punctum into the nasal cavity to prevent re-occlusion. A nasal endoscope was used to confirm and adjust the position of the stent (Figure 2d, 2e); subsequently, closure was initiated. The orbicularis oculi muscle and subcutaneous tissue were closed with 6-0 polydioxanone (PDS®), and the skin was closed with 6-0 polyvinylidene fluoride (ASFLEX®).

After 10 months, the tube was removed, and the patient underwent a final examination. A lacrimal syringe test was performed on the lower canaliculus. The lower lacrimal canaliculus allowed the passage of fluid. The epiphora was mild and not inconvenient; the patient did not wish to undergo any additional surgery.

## Discussion

In this report, two cases of common lacrimal canalicular lacerations were discussed. In the acute case, a bicanalicular silicone stent was successfully inserted into the nasolacrimal duct. In the patient with an old laceration, we converted the procedure from lacrimal canalicular anastomosis to Ex-DCR during exploration of the cut surface and inserted a monocanalicular silicon stent into the middle nasal cavity to achieve patency.

Several treatment methods have been reported for monocanalicular and bicanalicular lacerations [1,11-13]. The cut-off site was determined using a pigtail probe, a light guide, or by filling the lacrimal sac with viscoelastic material or air. Then, a bicanalicular silicon stent, a monocanalicular silicon stent, or two monostents were inserted into the lacrimal sac [7,14-18]. However, only a few reports have focused on the course of treatment for common lacrimal canalicular lacerations. Previously, reconstruction combined with endonasal dacryocystorhinostomy, and direct canalicular silicone tube insertion have been reported [6,9].

In the acute case, we identified the proximal cut surface of the common lacrimal canaliculus. We inserted a bicanalicular stent through the common lacrimal canaliculus into the nasolacrimal duct from the upper and lower puncta. Even if the cut surface is deep in the common lacrimal duct, with proper exploration, a bicanalicular stent can be inserted and treated in the same manner as that for upper and lower lacrimal canalicular lacerations.

In the case of an old laceration, we performed Ex-DCR and successfully placed a monocanalicular tube from the inferior lacrimal punctum into the nasal cavity. In old lacerations, it is recommended to use either canalicular anastomosis combined with stent intubation or DCR combined with stent intubation, depending on the wound condition and previous trauma. Given that a long time has passed since the injury and the wound condition is difficult to predict, it is better to plan surgery under general anesthesia. There is no risk of pain caused by previous scarring that makes local anesthesia ineffective. Further, it is easy to change to another surgical procedure, such as C-DCR, when no lacrimal canaliculus is found.

We could not completely reconstruct the upper and lower lacrimal canaliculi; however, in both cases, one of the upper and lower canaliculi was fully passed, and only a few subjective symptoms of tearing remained. In the postoperative period of the acute case, the tube was reinserted during a plastic surgery procedure for a zygomatic fracture a few days after the tube fell out; however, four months later, the inferior lacrimal duct was eventually occluded. The degree of trauma may have been severe enough to cause tear duct fibrosis. To address the old laceration, the tube was placed in a straight line from the inferior lacrimal punctum to the lacrimal sac and nasal cavity, and the syringing test was passed; however, owing to previous trauma, the inner side of the lower eyelid was facing toward the eyeball, and tear drainage was poor. We explained C-DCR as revision surgery to both patients; however, neither wanted to undergo the procedure because they experienced no serious interruptions to their daily lives. Postoperative management of C-DCR is difficult; therefore, inserting a lacrimal canalicular stent or treatment with DCR is a good option before performing C-DCR.

## Conclusions

This report describes an acute and a past case of common lacrimal canalicular lacerations. With proper preoperative evaluation and the approach, we could insert a bicanalicular stent in the acute case with a careful search for the common canaliculus, and we could treat an old laceration with Ex-DCR combined with canalicular stent insertion. Each approach is a good option before performing C-DCR.
